# Chronic Wasting Disease Prion Strain Emergence and Host Range Expansion

**DOI:** 10.3201/eid2309.161474

**Published:** 2017-09

**Authors:** Allen Herbst, Camilo Duque Velásquez, Elizabeth Triscott, Judd M. Aiken, Debbie McKenzie

**Affiliations:** University of Alberta, Edmonton, Alberta, Canada

**Keywords:** prions, prion diseases, prions and related diseases, host range, chronic wasting disease, pathogenicity, pathogen-host interactions, cervids, mice, zoonoses, Canada

## Abstract

Human and mouse prion proteins share a structural motif that regulates resistance to common chronic wasting disease (CWD) prion strains. Successful transmission of an emergent strain of CWD prion, H95^+^, into mice resulted in infection. Thus, emergent CWD prion strains may have higher zoonotic potential than common strains.

Chronic wasting disease (CWD) is a contagious prion disease of cervids that is spreading globally. CWD is enzootic in multiple cervid species, including deer and elk; the major foci of disease are Colorado/Wyoming (USA), Wisconsin/Illinois (USA), and Alberta/Saskatchewan (Canada). CWD is also present in captive cervids in South Korea and wild reindeer and moose in Norway (https://www.nwhc.usgs.gov/images/cwd/cwd_map.jpg). CWD results from the conformational transformation of the host-encoded cellular prion protein (PrP^C^) into protease-resistant, detergent-insoluble, β-sheet rich, amyloidogenic conformers, termed prions (PrP^CWD^). Within their conformation, prion strains encipher the information that directs the templated misfolding and aggregation of PrP^C^ molecules into additional prions (*1*).

Although the sequence homology of PrP among mammals is high, the ability of particular prion strains to cause disease in different species is determined by the conformational compatibility between a given strain and the host PrP^C^ (*2*). We previously identified 2 strains of CWD prion in white-tailed deer (*3*), Wisc-1 and H95^+^; these strains exhibit distinct biological properties in deer and transgenic cervidized mice. To ascertain the host range of different strains from cervids, we inoculated CWD prions isolated from experimentally infected deer with different *PRNP* genotypes (Q95G96 [wild type (wt)], S96/wt, H95/wt, and H95/S96) and from elk (CWD2 strain) into hamsters and mice. All isolates have been successfully transmitted into transgenic mice expressing wt cervid PrP and contain high titers of CWD prions (*3*).

Mice inoculated with H95^+^ CWD prions succumbed to clinical disease at 575 ± 47 or 692 ± 9 days, depending on the H95^+^ isolate (Table). Mice inoculated with Wisc-1 or elk CWD or uninfected deer homogenates were euthanized at day 708 after infection with no signs of prion disease. Clinical signs of H95^+^ CWD in C57Bl/6 mice included ataxia, lethargy, tail rigidity, and dermatitis. Protease-resistant PrP^CWD^ was present in all mice infected with H95^+^ prions and was not detected in mice infected with Wisc-1 or CWD2 (Technical Appendix).

**Table:** Results of CWD prion inoculation into rodents*

Recipient and CWD inocula	No.	PrP-res+		Incubation period, d
Clinical	Subclinical
Mice				
wt/wt	6	0	0	NA
S96/wt	6	0	0	NA
H95/wt	7	5	2	669, 671, 706, 706, 706
H95/S96	7	7	0	306, 593, 593, 593, 593, 673, 675
Elk	4	0	0	NA
Control	4	0	0	NA
Hamsters				
wt/wt	8	3	5	652, 653, 653
S96/wt	8	1	4	634
H95/wt	8	1	6	652
H95/S96	8	0	1	NA
Elk	8	2	2	673, 719
Control	8	0	0	NA

*Mice infected with CWD prions were observed for up to 708 d; hamsters infected with white-tailed deer and elk CWD prions were observed for 659 and 726 d, respectively. Control mice and hamsters were inoculated with brain homogenates from CWD-negative wt/wt deer. CWD, chronic wasting disease; NA, not applicable; PrP-res+, positive for proteinase-K–resistant prion protein; wt, wild type.

In contrast to mice, hamsters succumbed to clinical disease when inoculated with Wisc-1 CWD prions but were less susceptible to H95^+^ CWD prions (Table). Clinical signs of CWD in hamsters began with lethargy and, upon arousal, retrocollis; as the disease progressed, lethargy declined with increased dystonic movement including ataxia and tremors. Hyperesthesia was not observed. Subclinical disease (no clinical signs but PrP-res positive by Western blot) was observed in a subset of hamsters (Technical Appendix).

Successful interspecies prion transmission at the molecular level depends on the compatibility of the invading prion conformers and structural determinants imposed by host PrP^C^. One structural motif is the loop region between β sheet 2 and α helix 2 of PRP^C^ at aa 170–174 (Technical Appendix). Host species containing PrP^C^ molecules with a flexible β2-α2 loop (mice and humans) are hypothesized to be incompatible with prions derived from species containing a rigid loop (deer and elk) (*4*,*5*). Previous attempts to transmit CWD to mice have failed (*6*,*7*). Our data show that prions from a prototypic rigid-loop species (deer) can transmit to a flexible-loop species (mice). The transmission is strain dependent. H95^+^ overrides the conformational restriction imposed by the mouse PrP flexible loop that Wisc-1 and CWD2 cannot overcome, suggesting that the invading prion strain is a dominant contributor to the species/transmission barrier. How the N terminal amino acid polymorphism (Q95H) affects the conformation of PrP, altering the deer-to-mouse transmission barrier, is unknown. Further structural studies may clarify the effect of N terminal residues on β2-α2 loop rigidity.

Transmission of H95^+^ CWD prions to mice further confirms the value of specifying strain when defining species barriers. Experimental transmission of CWD prion into macaques and transgenic mice expressing human PrP suggests a considerable transmission barrier to CWD prions (although squirrel monkeys are susceptible), and human prion protein is converted inefficiently in vitro (*8*,*9*). Successful infection of a flexible-loop species (mice) with H95^+^ CWD raises concerns for the potential pathogenicity of H95^+^ prions to other flexible-loop species. Transmission studies with Wisc-1 and H95^+^ in transgenic humanized and bovinized mice are ongoing.

The increasing prevalence of CWD indicates selection for cervids with resistance alleles, such as S96 and H95. Genetic resistance to a given prion strain selects for the emergence of novel prion strains with altered properties such as H95^+^ and Nor98 (*3*,*10*). The iterative transmission of CWD prions to cervids with protective alleles of PrP^C^ and the consequent emergence of new CWD prion strains highlights the dynamics of the CWD panzootic and the value of characterizing the host range of emergent CWD prion strains.

Technical Appendix Additional data for study of emergent strains of chronic wasting disease prion and expanding host range.
