# Supplementary material for: Chronic Wasting Disease Prion Strain Emergence and Host Range Expansion
Source: Emerg Infect Dis. 2017 Sep;23(9):1598–600. doi: 10.3201/eid2309.161474 (PMC5572867; doi:10.3201/eid2309.161474)
Supplement: Technical Appendix — Additional data for study of emergent strains of chronic wasting disease prion and expanding host range. [file 16-1474-Techapp-s1.pdf]

Article DOI: https://doi.org/10.3201/eid2309.161474

Chronic Wasting Disease Prion Strain Emergence and Host Range Expansion

Technical Appendix

Additional Data

Brain homogenates from all mice (experimentally infected animals and uninfected controls) were analyzed for the presence of PrP-res. Proteinase K-resistant PrP was detected in all mice infected with $H95^+$ prions (Figure 1) demonstrating that $H95^+$ is 100% penetrant. PrP-res was not, however, detected in mice infected with the Wisc-1 strain from wt/wt or S96/wt deer or CWD2 from elk.

A similar analysis of brain homogenates from hamsters was also performed. Wisc-1 CWD was preferentially transmitted to hamsters upon primary passage (Figure 2). Wt/wt CWD caused clinical disease or subclinical accumulation of PrP-res in exposed hamsters. Transmission of S96/wt and H95/wt CWD prions resulted, primarily, in subclinical disease. Inoculation of elk CWD prions resulted in clinical disease in two out of five hamsters and subclinical disease in one animal. The H95/S96 isolate transmitted inefficiently with only one of eight hamsters having subclinical disease. This subclinical infection may be due to Wisc-1 in the H95/S96 deer isolate. Hamsters inoculated with uninfected deer brain homogenate did not show signs of prion disease nor accumulate PrP-res.

Secondary structure of host PrP influences the ability of a strain to propagate in a new host. Based on NMR studies, it has been hypothesized that amino acid variation at residues 170-175 (Figure 3) in PrP^C results in a loop structure between beta-sheet 2 and alpha-helix 2 (β2-α2) whose flexibility influences the outcome of interspecies transmissions (*1–7*). Mice and humans share a flexible loop sequence (SNQNN) whereas deer and elk possess a rigid loop (NNQNT). Multiple attempts to transmit CWD prions to mice have failed (*5–11*). The transmission of prions occurs more readily when the loop structures match, e.g. BSE transmits to humans and mice (*12*,*13*). Experimental alteration of the mouse flexible loop to a rigid loop facilitated CWD

transmission (*4*,*7*). Our data suggests emergent CWD strain H95$^+$ can overcome the conformational restriction imposed by the loop.

Methods and Materials

This study was conducted in accordance with the guidelines of the Canadian Council on Animal Care. The protocols used were approved by the Institutional Animal Care and Use Committees at the University of Alberta.

Brain homogenates were prepared from clinically-affected white-tailed deer (*Odocoileus virginianus*) of defined genotypes (*14*,*15*) or as a pool from three captive CWD-positive 132M/M elk (*Cervus canadensis*) (*16*,*17*), a kind gift from Catherine Graham. Four different white-tailed deer CWD isolates were used, one isolate was derived from a deer homozygous for the most common *PRNP* gene (wt = Q95G96) and three heterozygous deer (H95/wt, 96S/wt and H95/S96) expressing *PRNP* allele variants. These isolates have been extensively characterized in transgenic mice (*15*). Weanling C57Bl/6 mice were intracerebrally inoculated with 30 µl of 1% brain homogenate. Weanling Syrian Golden hamsters (*Mesocricetus auratus*) were intracerebrally inoculated with 50 µl of 10% brain homogenates (elk CWD was passaged at 1% brain homogenate). Mock-infected controls received brain homogenate from unaffected, PrP-res negative deer. Mice and hamsters were monitored for onset of clinical signs and disease progression. Individual incubation periods are expressed as the number of days post-inoculation (dpi) and were calculated from the time that the mice were inoculated until the time that clinical disease was established. Experiments were terminated at 708 dpi (mice) and 659 dpi (hamsters); all remaining animals were euthanized and assayed for PrP-res.

Brains were collected from mice and hamsters, flash frozen and stored at -80 until analysis. Tissues were homogenized to 10% (wt/vol) in sterile water with a tissue disruptor (Omniprep) and disposable homogenization tubes and beads. PrP-res was detected following proteinase K digestion and immunoblotting as previously described (*15*). Blots were probed with antibodies SAF83 (mice; Cayman Chemical, Michigan) or 3F4 (hamsters; a kind gift from Richard Rubenstein) monoclonal antibodies.

References

1. Gossert AD, Bonjour S, Lysek DA, Fiorito F, Wüthrich K. Prion protein NMR structures of elk and of mouse/elk hybrids. Proc Natl Acad Sci U S A. 2005;102:646–50. PubMed http://dx.doi.org/10.1073/pnas.0409008102

2. Gorfe AA, Caflisch A. Ser170 controls the conformational multiplicity of the loop 166-175 in prion proteins: implication for conversion and species barrier. FASEB J. 2007;21:3279–87. PubMed http://dx.doi.org/10.1096/fj.07-8292com

3. Sigurdson CJ, Nilsson KP, Hornemann S, Heikenwalder M, Manco G, Schwarz P, et al. De novo generation of a transmissible spongiform encephalopathy by mouse transgenesis. Proc Natl Acad Sci U S A. 2009;106:304–9. PubMed http://dx.doi.org/10.1073/pnas.0810680105

4. Sigurdson CJ, Nilsson KP, Hornemann S, Manco G, Fernández-Borges N, Schwarz P, et al. A molecular switch controls interspecies prion disease transmission in mice. J Clin Invest. 2010;120:2590–9. PubMed http://dx.doi.org/10.1172/JCI42051

5. Bett C, Fernández-Borges N, Kurt TD, Lucero M, Nilsson KP, Castilla J, et al. Structure of the β2-α2 loop and interspecies prion transmission. FASEB J. 2012;26:2868–76. PubMed http://dx.doi.org/10.1096/fj.11-200923

6. Kurt TD, Bett C, Fernández-Borges N, Joshi-Barr S, Hornemann S, Rülicke T, et al. Prion transmission prevented by modifying the β2-α2 loop structure of host PrPC. J Neurosci. 2014;34:1022–7. PubMed http://dx.doi.org/10.1523/JNEUROSCI.4636-13.2014

7. Kurt TD, Jiang L, Fernández-Borges N, Bett C, Liu J, Yang T, et al. Human prion protein sequence elements impede cross-species chronic wasting disease transmission. J Clin Invest. 2015;125:1485–96. PubMed http://dx.doi.org/10.1172/JCI79408

8. Browning SR, Mason GL, Seward T, Green M, Eliason GA, Mathiason C, et al. Transmission of prions from mule deer and elk with chronic wasting disease to transgenic mice expressing cervid PrP. J Virol. 2004;78:13345–50. PubMed http://dx.doi.org/10.1128/JVI.78.23.13345-13350.2004

9. Raymond GJ, Raymond LD, Meade-White KD, Hughson AG, Favara C, Gardner D, et al. Transmission and adaptation of chronic wasting disease to hamsters and transgenic mice: evidence for strains. J Virol. 2007;81:4305–14. PubMed http://dx.doi.org/10.1128/JVI.02474-06

10. Meyerett C, Michel B, Pulford B, Spraker TR, Nichols TA, Johnson T, et al. In vitro strain adaptation of CWD prions by serial protein misfolding cyclic amplification. Virology. 2008;382:267–76. PubMed http://dx.doi.org/10.1016/j.virol.2008.09.023

11. Trifilo MJ, Ying G, Teng C, Oldstone MB. Chronic wasting disease of deer and elk in transgenic mice: oral transmission and pathobiology. Virology. 2007;365:136–43. PubMed http://dx.doi.org/10.1016/j.virol.2007.03.032

12. Bruce ME, Will RG, Ironside JW, McConnell I, Drummond D, Suttie A, et al. Transmissions to mice indicate that 'new variant' CJD is caused by the BSE agent. Nature. 1997;389:498–501. PubMed http://dx.doi.org/10.1038/39057

13. Hill AF, Desbruslais M, Joiner S, Sidle KC, Gowland I, Collinge J, et al. The same prion strain causes vCJD and BSE. Nature. 1997;389:448–50, 526. PubMed http://dx.doi.org/10.1038/38925

14. Johnson CJ, Herbst A, Duque-Velasquez C, Vanderloo JP, Bochsler P, Chappell R, et al. Prion protein polymorphisms affect chronic wasting disease progression. PLoS One. 2011;6:e17450. PubMed http://dx.doi.org/10.1371/journal.pone.0017450

15. Duque Velásquez C, Kim C, Herbst A, Daude N, Garza MC, Wille H, et al. Deer Prion Proteins Modulate the Emergence and Adaptation of Chronic Wasting Disease Strains. J Virol. 2015;89:12362–73. PubMed http://dx.doi.org/10.1128/JVI.02010-15

16. Pushie MJ, Shaykhutdinov R, Nazyrova A, Graham C, Vogel HJ. An NMR metabolomics study of elk inoculated with chronic wasting disease. J Toxicol Environ Health A. 2011;74:1476–92. PubMed http://dx.doi.org/10.1080/15287394.2011.618977

17. Angers RC, Kang HE, Napier D, Browning S, Seward T, Mathiason C, et al. Prion strain mutation determined by prion protein conformational compatibility and primary structure. Science. 2010;328:1154–8. PubMed http://dx.doi.org/10.1126/science.1187107

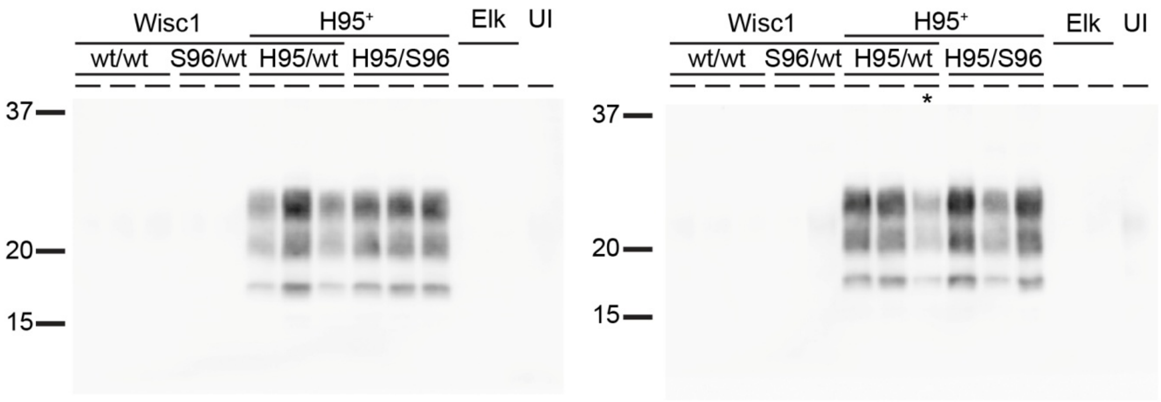

Figure 1. Representative western blot analysis of PrP-res on primary passage of CWD in mice. Each lane is an individual mouse brain homogenate sample treated with 50 µg/ml of proteinase K; equivalent volumes were loaded in each well. Only mice infected with H95+ isolates accumulated PK-resistant PrP. UI is a brain homogenate from a mouse inoculated with CWD negative wt/wt deer brain homogenate. Blots were probed with monoclonal antibody SAF83 at a 1:10,000 dilution. An asterisk denotes a subclinical mouse.

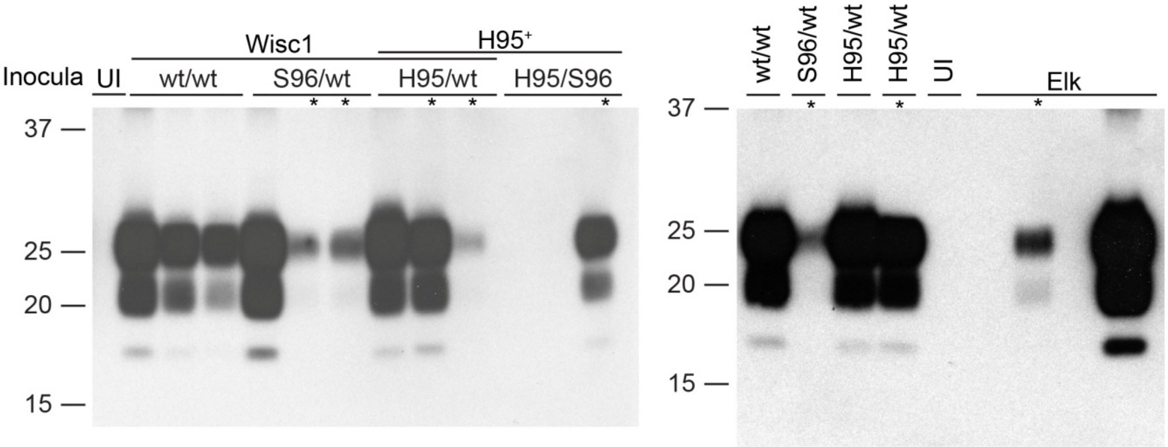

Figure 2. Representative western blot analysis of PrP-res on primary passage of CWD prions into Syrian golden hamsters. A. Brain homogenates from each passage line (10 µl of 10% brain homogenate) were treated with 100 µg/ml of proteinase K and 10 µl of each digested sample was loaded. One of 8 hamsters inoculated with H95/S96 CWD accumulated PK-resistant PrP. UI is a brain homogenate from a mock-infected hamster. Blots were probed with monoclonal antibody 3F4 at a 1:10,000 dilution. Asterisks denote subclinically affected hamsters.

```
         10        20        30        40        50        60
--MANLGCWMLVLFVATWSDLGLCKKRPKPGG-WNTGGSRYPGQGSPGGNRYPPQGGGGWGQPHG Homo sapiens
--MANLGYWLLALFVTMWTDVGLCKKRPKPGG-WNTGGSRYPGQGSPGGNRYPPQGG-TWGQPHG Mus musculus
MVKSHIGSWILVLFVAMWSDVGLCKKRPKPGGGWNTGGSRYPGQGSPGGNRYPPQGGGGWGQPHG Odocoileus virginianus wt
MVKSHIGSWILVLFVAMWSDVGLCKKRPKPGGGWNTGGSRYPGQGSPGGNRYPPQGGGGWGQPHG Odocoileus virginianus H95
MVKSHIGSWILVLFVAMWSDVGLCKKRPKPGGGWNTGGSRYPGQGSPGGNRYPPQGGGGWGQPHG Odocoileus virginianus S96
MVKSHIGSWILVLFVAMWSDVGLCKKRPKPGGGWNTGGSRYPGQGSPGGNRYPPQGGGGWGQPHG Cervus canadensis
--MANLSYWLLALFVAMWTDVGLCKKRPKPGG-WNTGGSRYPGQGSPGGNRYPPQGGGTWGQPHG Mesocricetus auratus

      70        80         90       100       110       120
GGWGQPHGGGWGQPHGGGWGQPHGGG-WGQGGGTHSQWNKPSKPKTNMKHMAGAAAAGAVVGGLG Homo sapiens
GGWGQPHGGSWGQPHGGSWGQPHGGG-WGQGGGTHNQWNKPSKPKTNLKHVAGAAAAGAVVGGLG Mus musculus
GGWGQPHGGGWGQPHGGGWGQPHGGGGWGQGG-THSQWNKPSKPKTNMKHVAGAAAAGAVVGGLG Odocoileus virginianus wt
GGWGQPHGGGWGQPHGGGWGQPHGGGGWGHGG-THSQWNKPSKPKTNMKHVAGAAAAGAVVGGLG Odocoileus virginianus H95
GGWGQPHGGGWGQPHGGGWGQPHGGGGWGQSG-THSQWNKPSKPKTNMKHVAGAAAAGAVVGGLG Odocoileus virginianus S96
GGWGQPHGGGWGQPHGGGWGQPHGGGGWGQGG-THSQWNKPSKPKTNMKHVAGAAAAGAVVGGLG Cervus canadensis
GGWGQPHGGGWGQPHGGGWGQPHGGG-WGQGGGTHNQWNKPSKPKTNMKHMAGAAAAGAVVGGLG Mesocricetus auratus

 130       140       150       160       170       180       190
GYMLGSAMSRPIIHFGSDYEDRYYRENMHRYPNQVYYRPMDEYSNQNNFVHDCVNITIKQHTVTT Homo sapiens
GYMLGSAMSRPMIHFGNDWEDRYYRENMYRYPNQVYYRPVDQYSNQNNFVHDCVNITIKQHTVTT Mus musculus
GYMLGSAMSRPLIHFGNDYEDRYYRENMYRYPNQVYYRPVDQYNNQNTFVHDCVNITVKQHTVTT Odocoileus virginianus wt
GYMLGSAMSRPLIHFGNDYEDRYYRENMYRYPNQVYYRPVDQYNNQNTFVHDCVNITVKQHTVTT Odocoileus virginianus 95H
GYMLGSAMSRPLIHFGNDYEDRYYRENMYRYPNQVYYRPVDQYNNQNTFVHDCVNITVKQHTVTT Odocoileus virginianus 96S
GYMLGSAMSRPLIHFGNDYEDRYYRENMYRYPNQVYYRPVDQYNNQNTFVHDCVNITVKQHTVTT Cervus canadensis
GYMLGSAMSRPMMHFGNDWEDRYYRENMNRYPNQVYYRPVDQYNNQNNFVHDCVNITIKQHTVTT Mesocricetus auratus

       200       210       220         230       240       250
TTKGENFTETDVKMMERVVEQMCITQYERESQAYYQ--RGSSMVLFSSPPVILLISFLIFLIVG  Homo sapiens
TTKGENFTETDVKMMERVVEQMCVTQYQKESQAYYDGRRSSSTVLFSSPPVILLISFLIFLIVG  Mus musculus
TTKGENFTETDIKMMERVVEQMCITQYQRESQAYYQ--RGASVILFSSPPVILLISFLIFLIVG  Odocoileus virginianus wt
TTKGENFTETDIKMMERVVEQMCITQYQRESQAYYQ--RGASVILFSSPPVILLISFLIFLIVG  Odocoileus virginianus H95
TTKGENFTETDIKMMERVVEQMCITQYQRESQAYYQ--RGASVILFSSPPVILLISFLIFLIVG  Odocoileus virginianus S96
TTKGENFTETDIKMMERVVEQMCITQYQRESEAYYQ--RGASVILFSSPPVILLISFLIFLIVG  Cervus canadensis
TTKGENFTETDIKIMERVVEQMCTTQYQKESQAYYDGRRSS-AVLFSSPPVILLISFLIFLMVG  Mesocricetus auratus
```

Figure 3. Alignment of PrP amino acid sequences from humans, cervids and rodents. Numbering is based on the human sequence. Deer polymorphisms at 95, 96 (human residues 91, 92 respectively) and residues forming the β2-α2 loop are boxed.
